# Cognitive status and demographics modify the association between subjective cognition and amyloid

**DOI:** 10.1002/acn3.52209

**Published:** 2024-10-23

**Authors:** Corey J. Bolton, Omair A. Khan, Dandan Liu, Sydney Wilhoite, Logan Dumitrescu, Amalia Peterson, Kaj Blennow, Henrik Zetterberg, Timothy J. Hohman, Angela L. Jefferson, Katherine A. Gifford

**Affiliations:** ^1^ Vanderbilt Memory and Alzheimer's Center Vanderbilt University Medical Center Nashville Tennessee USA; ^2^ Department of Medicine Vanderbilt University Medical Center Nashville Tennessee USA; ^3^ Department of Biostatistics Vanderbilt University Medical Center Nashville Tennessee USA; ^4^ Department of Neurology Vanderbilt University Medical Center Nashville Tennessee USA; ^5^ Department of Psychiatry and Neurochemistry, Institute of Neuroscience and Physiology The Sahlgrenska Academy at University of Gothenburg Mölndal Sweden; ^6^ Clinical Neurochemistry Lab Sahlgrenska University Hospital Mölndal Sweden; ^7^ Department of Neurodegenerative Disease UCL Institute of Neurology, Queen Square London UK; ^8^ UK Dementia Research Institute at UCL London UK; ^9^ Hong Kong Center for Neurodegenerative Diseases Hong Kong China; ^10^ Wisconsin Alzheimer's Disease Research Center, University of Wisconsin School of Medicine and Public Health University of Wisconsin‐Madison Madison Wisconsin USA

## Abstract

**Objective:**

This study examined the effect of cognitive status, education, and sex on the association between subjective cognitive decline (SCD) and Alzheimer's disease (AD) biomarkers in non‐demented older adults.

**Methods:**

Vanderbilt Memory and Aging Project participants (*n* = 129), dementia or stroke free, completed fasting lumbar puncture, SCD assessment, and cognitive assessment. Cerebrospinal fluid (CSF) biomarkers for AD were analyzed. Linear regression models related SCD to CSF AD biomarkers and follow‐up models assessed interactions of *SCD × cognitive status, sex, reading level*, and *education* on AD biomarkers.

**Results:**

In main effect models, higher SCD was associated with more amyloidosis (*p*‐values <0.004). SCD was not associated with tau, p‐tau, or neurofilament light (NFL) levels (*p‐*values >0.38). SCD score interacted with cognitive status (*p* < 0.02), sex (*p* = 0.03), and education (*p*‐values *<*0.005) on amyloidosis. In stratified models, higher SCD was associated with more amyloid in cognitively unimpaired (*p*‐values <0.003), men (*p* = 0.0003), and higher education. No *SCD score × reading‐level* interaction was found (*p*‐values >0.51) though SCD related to amyloid markers in the higher reading‐level group (*p*‐values <0.004).

**Interpretation:**

Higher SCD was associated with greater cerebral amyloid accumulation, one of the earliest pathological AD changes. SCD appears most useful in detecting early AD‐related brain changes prior to objective cognitive impairment, in men, and those with higher quantity and quality of education and highlight the importance of considering these factors.

## Introduction

Alzheimer's disease (AD) is a public health crisis that will only continue to worsen as the population ages.^1^ Novel treatments for AD require initiation prior to the onset of dementia, highlighting the need for early detection in individuals who are cognitively unimpaired or only mildly symptomatic.^2^ Current methods of identifying underlying AD pathology in non‐demented individuals, such as positron emission tomography imaging and lumbar puncture, can be expensive and would place an unsustainable burden on the health care system if implemented widely. Screening patients to identify those at elevated risk is one way to dramatically reduce the cost associated with early identification of AD pathology.^3^ Screening measures that are efficient and cost‐effective are essential to sustain the current needs for early identification in AD.

One efficient and inexpensive method of screening and identifying patients at increased risk of AD is through utilizing measures of subjective cognitive decline (SCD). Higher levels of SCD have been associated with cognitive decline,^4^, ^5^ hippocampal atrophy,^6^ changes in cerebral blood flow,^6^ and progression to MCI and dementia.^7^ Despite the utility of SCD in predicting AD‐related brain and clinical changes, there are numerous factors beyond AD that can contribute to SCD and many clinical and demographic factors that may modify the ability of SCD to predict AD‐related changes. For example, as individuals develop cognitive impairment due to AD, a common symptom is anosognosia, a lack of awareness of their deficits, which could certainly impact their self‐report of cognitive symptoms. Addtionally, SCD has been more strongly associated with clinical decline in women than in men,^8^ and in individuals with higher levels of education compared to those with lower levels of education.^7^ SCD has also been associated with cerebrospinal fluid (CSF) biomarkers of AD^9^; however, this association is inconsistent,^10^ possibly due to demographic differences. Despite recognition that clinical and demographic factors may influence AD biology and clinical manifestation, there is a paucity of work examining the specific factors and nature of the effect modification of these factors on the ability of SCD to predict underlying AD pathology in non‐demented patients.

This study seeks to examine the associations between a novel SCD measure and CSF biomarkers of AD in non‐demented older adults, and to determine the effect of cognitive status and common demographic factors on this association. We hypothesize that SCD will be more strongly associated with CSF biomarkers of AD in individuals who are cognitively unimpaired, due to the potential for anosognosia in those with objective cognitive impairment. Based on past work,^7^, ^8^, ^9^ we are focusing on sex and education as potentially modifying demographic factors. We hypothesize that this novel SCD measure will be associated with CSF biomarkers of AD and that these associations will be stronger in women. We will also investigate two different markers of education, including years of education completed and a single‐word reading metric as a proxy for educational quality. We hypothesize SCD and CSF biomarker associations will be stronger in individuals with more years of education and greater educational quality. We aim to identify which factors influence the association of SCD and underlying AD pathology to aid in identifying patients in whom novel interventions may be most beneficial.

## Methods

### Cohort

Participants were drawn from the baseline cohort of the Vanderbilt Memory and Aging Project, a longitudinal study investigating vascular health and brain health among aging adults.^11^ Inclusion criteria required participants to be age 60 or older, speak English, have adequate visual and auditory acuity, and have a reliable study partner. To determine study eligibility, participants completed a medical history review, clinical interview, and neuropsychological assessment. Cognitive diagnosis was determined by consensus, including cognitively unimpaired, early mild cognitive impairment (eMCI; defined as a Clinical Dementia Rating Scale of 0 and mild objective cognitive impairment in 1 cognitive domain or Clinical Dementia Rating Scale of 0.5 and minimal objective cognitive impairment),^12^ or MCI based on the National Institute on Aging/Alzheimer's Association Workgroup clinical criteria.^13^ Participants were excluded for magnetic resonance imaging (MRI) contraindication, history of neurological disease (e.g., dementia and stroke), major psychiatric illness, heart failure, severe head injury (loss of consciousness ≥5 min), and systemic or terminal illness (e.g., cancer) that could affect follow‐up participation. At study enrollment, participants completed a comprehensive evaluation, including but not limited to physical and frailty examination, fasting blood draw, clinical interview, SCD module, echocardiogram, brain MRI, and optional lumbar puncture. Participants were excluded from the current analyses for missing baseline SCD, covariate, or CSF data.

The protocol was approved by the Vanderbilt University Medical Center Institutional Review Board, and written informed consent was obtained from all participants prior to data collection. Due to participant consent limitations in data sharing, a subset of data is available for purposes of reproducing the results or procedures. These data, analytic methods, and study materials can be obtained by contacting the corresponding author.

### SCD questionnaire

Participants completed four questionnaires assessing SCD: the Everyday Cognition Questionnaire,^14^ the Memory Functioning Questionnaire,^15^ the Cognitive Difficulties Scale,^16^ and the Cognitive Changes Questionnaire.^17^ Items from these questionnaires were reduced into a 45‐item questionnaire (the Vanderbilt SCD Questionnaire) using psychometric methods including item response theory and computerized adaptive testing.^18^ Scores on this measure range from 38 to 192, with higher scores indicating more SCD. The current study considered SCD score as a continuous measure, as opposed to a dichotomized diagnostic status. This measure was not used to determine study eligibility or cognitive status.

### Lumbar puncture and biochemical analyses

Participants completed an optional fasting lumbar puncture at study enrollment. CSF was collected with polypropylene syringes using a Sprotte 25‐gauge spinal needle in an intervertebral lumbar space. Samples were immediately mixed and centrifuged, and supernatants were aliquoted in 0.5 mL polypropylene tubes and stored at −80°C. Samples were analyzed in batch using commercially available enzyme‐linked immunosorbent assays (Fujirebio, Ghent, Belgium) to determine the levels of amyloid‐β_1‐42_ (Aβ_42_; INNOTEST^®^ β‐AMYLOID_(1–42)_), Aβ_42_, and Aβ_40_ to calculate the Aβ_42/40_ ratio (Aβ Triplex Assay, Meso Scale Discovery), phosphorylated tau (p‐tau; INNOTEST^®^ PHOSPHO‐TAU_(181P)_), and total tau (t‐tau; INNOTEST^®^ hTAU). P‐tau was measured by tagging a tau phosphorylation site at threonine 181. Neurofilament light (NfL) was measured using a commercially available enzyme‐linked immunosorbent assay (Uman Diagnostics). Board‐certified laboratory technicians processed data blinded to clinical information, as previously described.^19^ Intra‐assay coefficients of variation were <10%.

### Reading‐level assessment

Reading level was assessed at eligibility using the Wide Range Achievement Test 3rd edition (WRAT‐III) Reading subtest.^20^ Scores on this measure range from 0 to 57, with 0–41 representing approximately below high school reading level, 42–47 representing high school reading level, and 48–57 representing post‐high school reading level. For stratified analyses, reading level was dichotomized by a median split. This test is a commonly used measure to estimate premorbid intelligence and education quality.^21^

### Covariates

The current study adjusted for age, sex, education, race/ethnicity, *APOE‐ ε*4 status, cognitive status, and score on the Geriatric Depression Scale (GDS).^22^ APOE genotyping was performed using a TaqMan assay on DNA extracted from whole‐blood samples,^11^ and APOE‐ε4 carrier status was defined as positive (ε2/ε4, ε3/ε4, ε4/ε4) or negative (ε2/ε2, ε2/ε3, ε3/ε3). The following questions related to SCD/cognition were excluded from the GDS score, as these data are likely to confound analyses with SCD as our predictor: “Do you feel you have more problems with your memory than most?” “Do you have trouble concentrating?” “Is it easy for you to make decisions?” and “Is your mind as clear as it used to be?”

### Analytic plan

Linear regression models related SCD to CSF AD biomarkers (Aβ_42_, Aβ_42/40_ ratio, tau, p‐tau, and NfL), adjusting for age, sex, education, race/ethnicity, *APOE‐ ε*4 status, cognitive status, and GDS score. Follow‐up models assessed *SCD × cognitive status, SCD × sex*, *SCD × reading level*, and *SCD × education* interactions (with all covariates from initial models) on AD biomarkers with subsequent models stratified by cognitive status (cognitively unimpaired, MCI), sex (male, female), reading‐level split at median (lower half, upper half), and education (lowest tertile, highest tertile), respectively.

Sensitivity analyses excluded predictor or outcome values >4 standard deviations from the group mean to determine if outliers influenced results. Multiple comparison correction was performed across outcomes per model using a false discovery rate based on Benjamini–Hochberg's procedure. Analyses were performed using R 3.5.2 (www.r‐project.org) and significance was set a priori at *p* < 0.05.

## Results

### Participant characteristics

Participants included 129 adults ages 61–90 (33% MCI, 28% female, 94% non‐Hispanic White, 31% *APOE*‐ε4 carriers, 16 ± 3 years of education). SCD was significantly correlated with CSF Aβ_42_ (*r* = −0.30, *p* = 0.0006) and Aβ_42/40_ (*r* = −0.28, *p* = 0.001), but not other outcomes (*p*‐values >0.06). See Table 1 for participant characteristics for the entire sample and stratified by cognitive status.

**Table 1:** Participant characteristics.

	Combined (*n* = 129)	Normal (*n* = 72)	eMCI (*n* = 14)	MCI (*n* = 43)	*p*‐value^b^
Age, years	72.9 ± 7	72.5 ± 6.6	72.7 ± 5.8	73.6 ± 6.4	0.8
Sex, % female	28	25	21	35	0.44
Education, years	16.1 ± 3	16.6 ± 3	15.9 ± 3	15.2 ± 3	**0.04**
Race, % non‐Hispanic White	94	94	93	93	0.94
APOE *ε*4, % carrier	31	29	7	42	**0.05**
GDS score^a^	2.2 ± 2.6	2.1 ± 2.8	0.9 ± 1.7	3.0 ± 2.5	**0.002**
WRAT reading score	51.0 ± 5.2	51.9 ± 4.2	49.1 ± 3.7	49.9 ± 5.3	**0.02**
SCD score	62.3 ± 25.3	53 ± 18	75 ± 26	74 ± 21	**<0.001**
Aβ_42_, pg/mL	553.6 ± 301.4	598 ± 260	733 ± 349	421 ± 199	**<0.001**
Aβ_42/40_	0.89 ± 0.4	0.94 ± 0.3	1.06 ± 0.3	0.73 ± 0.3	**<0.001**
Tau, pg/mL	422.8 ± 223.0	374 ± 174	429 ± 129	503 ± 259	**0.02**
P‐tau, pg/mL	60.7 ± 26.8	56 ± 21	64 ± 18	68 ± 30	0.05
NfL, pg/mL	1098.0 ± 574.4	963 ± 467	1091 ± 482	1323 ± 774	**0.005**

Values denoted as mean ± standard deviation or frequency. Bold font indicates *p*‐value <0.05.

Aβ, amyloid beta; GDS, Geriatric Depression Scale; MCI, mild cognitive impairment; NfL, neurofilament light; p‐tau, phosphorylated tau; SCD, subjective cognitive decline; WRAT, Wide Range Achievement Test 3rd edition.

^a^ Minus points for cognition.

^b^ Kruskal–Wallis test was used for continuous variables, and Pearson's chi‐squared test was used for categorical variables.

### SCD and CSF biomarkers

**Figure 1:**
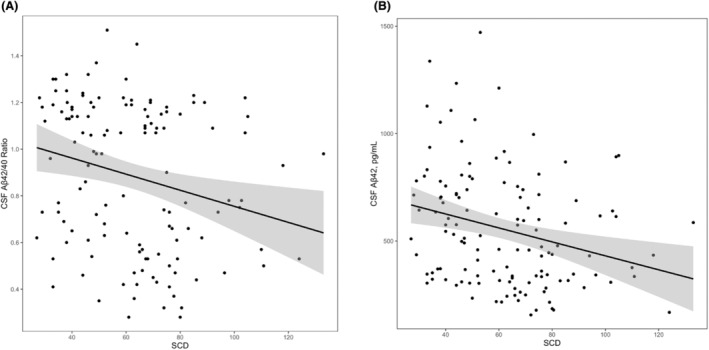
Associations between subjective cognitive decline and cerebrospinal fluid levels of amyloid‐β_42_ and amyloid‐β_42/40_ ratio. Lines reflect CSF biomarker values corresponding to SCD levels. Shading reflects 95% confidence interval. (A) Associations between SCD and CSF Aβ_42/40_ ratio, *β* = −0.004, *p* = 0.004. (B) Associations between SCD and CSF Aβ_42_, *β* = −3.34, *p* = 0.003. Aβ, amyloid beta; SCD, subjective cognitive decline.

Greater SCD was associated with lower CSF Aβ_42_ (*β* = −3.34, *p* = 0.003) and Aβ_42/40_ (*β* = −0.004, *p* = 0.004) but was not associated with CSF levels of tau, p‐tau, and NfL (*p*‐values >0.38). These results persisted after FDR correction and were largely unchanged in sensitivity analyses excluding outliers (see Table S1). See Table 2 for results and Figure 1 for illustrations.

**Table 2:** SCD associations with CSF biomarkers.

	*β*	95% CI	*p*
Aβ_42_, pg/mL	−3.34	−5.54, −1.15	**0.003** *
Aβ_42/40_	−0.004	−0.006, −0.001	**0.004** *
Tau, pg/mL	0.84	−1.06, 2.75	0.38
P‐tau, pg/mL	0.08	−0.14, 0.31	0.46
NfL, pg/mL	2.04	−3.62, 7.70	0.48

Models were adjusted for age, sex, education, race/ethnicity, *APOE*‐ε4 status, cognitive status, and GDS. *β* indicates the degree of change in outcomes per 1 unit increase in SCD. Bold font indicates *p*‐value <0.05.

Aβ, amyloid beta; APOE, apolipoprotein E; CSF, cerebrospinal fluid; FDR, false discovery rate; GDS, Geriatric Depression Scale; NfL, neurofilament light; p‐tau, phosphorylated tau; SCD, subjective cognitive decline.

* FDR‐adjusted *p*‐value <0.05.

### *SCD × cognitive status* interactions on CSF biomarkers

**Figure 2:**
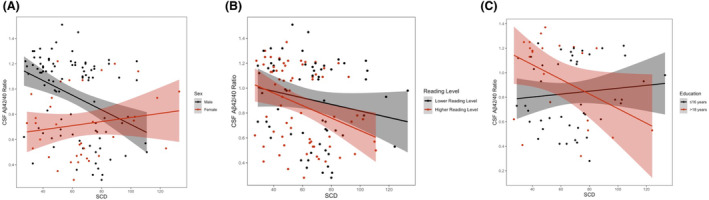
Subjective cognitive decline × sex, reading level, and education interactions on cerebrospinal fluid amyloid‐β_42/40_ ratio. Lines reflect CSF Aβ_42/40_ values corresponding to SCD levels. Shading reflects 95% confidence interval. (A) Associations between SCD and CSF Aβ_42/40_ ratio, stratified by sex; males *b* = −0.006, *p* = 0.0003, females *b* = 0.02, *p* = 0.48. (B) Associations between SCD and CSF Aβ_42/40_ ratio, stratified by reading level; lower reading level *β* = −0.003, *p* = 0.12, higher reading level *β* = −0.005, *p* = 0.004. (C) Associations between SCD and CSF Aβ_42/40_ ratio, stratified by education; ≤16 years *β* = 0.002, *p* = 0.28, >18 years *β* = −0.006, *p* = 0.23. Aβ, amyloid beta; SCD, subjective cognitive decline.

SCD interacted with cognitive status on Aβ_42_ (*β* = 5.75, *p* = 0.01) and Aβ_42/40_ ratio (*β* = 0.006, *p* = 0.02) but not on any other CSF biomarkers (*p*‐values >0.05). In stratified analyses, SCD was associated with Aβ_42_ (*β* = −5.39, *p* = 0.003) and Aβ_42/40_ (*β* = −0.006, *p* = 0.001) in cognitively unimpaired participants, but not in participants with MCI (*p*‐values >0.69). These results persisted after outlier exclusion and were largely unchanged following FDR correction (*SCD × cognitive status* interaction on Aβ_42/40_ ratio was mildly attenuated (*p* = 0.05)). See Table 3 for results and Figure 2 for illustrations.

**Table 3:** SCD and CSF biomarkers: interaction and stratified models.

	*β*	95% CI	*p*	*β*	95% CI	*p*	*β*	95% CI	*p*
SCD × diagnosis interactions				Cognitively unimpaired (*n* = 72)			MCI (*n* = 43)		
Aβ_42_, pg/mL	5.75	1.48, 10.01	**0.009** *	−5.39	−8.83, −1.96	**0.003** *	0.35	−2.73, 3.43	0.82
Aβ_42/40_	0.006	0.001, 0.01	**0.02**	−0.006	−0.01, −0.003	**0.0005** *	−0.0009	−0.006, 0.004	0.69
Tau, pg/mL	−2.21	−6.24, 1.81	0.28	1.70	−0.73, 4.13	0.17	0.87	−3.43, 5.18	0.68
P‐tau, pg/mL	−0.28	−0.76, 0.20	0.25	0.19	−0.11, 0.49	0.21	0.04	−0.46, 0.55	0.87
NfL, pg/mL	11.62	−0.09, 23.33	0.05	−5.38	−11.78, 1.01	0.10	11.46	−2.16, 25.09	0.10
SCD × sex interactions				Men (*n* = 93)			Women (*n* = 36)		
Aβ_42_, pg/mL	3.12	−0.59, 6.80	0.10	−5.11	−7.94, −2.29	**0.0005** *	1.59	−2.40, 5.58	0.42
Aβ_42/40_	0.005	0.0006, 0.009	**0.03**	−0.006	−0.009, −0.003	**0.0003** *	0.002	−0.003, 0.007	0.48
Tau, pg/mL	−2.27	−5.48, 0.94	0.16	1.78	−0.39, 3.95	0.11	0.65	−4.36, 5.66	0.79
P‐tau, pg/mL	−0.26	−0.64, 0.13	0.19	0.16	−0.11, 0.43	0.25	0.06	−0.50, 0.63	0.82
NfL, pg/mL	−3.29	−12.82, 6.25	0.50	3.86	−3.43, 11.15	0.29	−0.51	−12.19, 11.16	0.93
SCD × reading‐level interactions				Lower half (*n* = 61)			Upper half (*n* = 68)		
Aβ_42_, pg/mL	−1.86	−8.22, 4.50	0.56	−2.30	−5.75, 1.15	0.19	−5.70	−9.10, −2.29	**0.001** *
Aβ_42/40_	−0.002	−0.010, 0.005	0.84	−0.003	−0.007, 0.0008	0.12	−0.005	−0.009, −0.002	**0.004** *
Tau, pg/mL	1.97	−3.50, 7.45	1.00	0.68	−1.48, 2.84	0.53	1.63	−1.67, 4.92	0.33
P‐tau, pg/mL	0.18	−0.48, 0.84	0.97	0.09	−0.17, 0.35	0.50	0.11	−0.29, 0.51	0.60
NfL, pg/mL	−7.19	−23.55, 9.17	0.51	3.54	−6.72, 13.80	0.49	2.78	−2.72, 8.29	0.32
SCD × education interactions				Lowest tertile (*n* = 45)			Highest tertile (*n* = 22)		
Aβ_42_, pg/mL	−0.72	−1.21, −0.22	**0.005** *	1.25	−2.32, 4.81	0.48	−4.36	−10.35, 1.63	0.14
Aβ_42/40_	−0.0009	−0.001, −0.0004	**0.001** *	0.002	−0.002, 0.005	0.28	−0.006	−0.02, 0.005	0.23
Tau, pg/mL	0.27	−0.17, 0.70	0.23	0.18	−3.33, 3.70	0.92	1.12	−5.68, 7.91	0.73
P‐tau, pg/mL	0.03	−0.02, 0.08	0.24	0.03	−0.37, 0.43	0.82	0.10	−0.83, 1.04	0.81
NfL, pg/mL	−0.01	−1.31, 1.28	0.98	7.52	−0.92, 15.95	0.08	0.22	−10.66, 11.10	0.97

Models were adjusted for age, sex, education, race/ethnicity, *APOE*‐ε4 status, cognitive status, and GDS. *β* indicates the degree of change in outcomes per 1 unit increase in SCD. Bold font indicates *p*‐value <0.05.

Aβ, amyloid beta; APOE, apolipoprotein E; FDR, false discovery rate; GDS, Geriatric Depression Scale; NfL, neurofilament light; p‐tau, phosphorylated tau; SCD, subjective cognitive decline.

* FDR‐adjusted *p*‐value <0.05.

### *SCD × sex* interactions on CSF biomarkers

SCD interacted with sex on Aβ_42/40_ (*β* = 0.005, *p* = 0.03) but not on any other CSF biomarkers (*p*‐values >0.10). In stratified analyses, SCD was associated with Aβ_42/40_ in men (*β* = −0.006, *p* = 0.0003), but not in women (*p* = 0.48). These results were largely unchanged in sensitivity analyses excluding outliers (see Table S1) but the overall interaction was attenuated after FDR correction (*p* = 0.15). See Table 3 for results and Figure 2 for illustrations.

### *SCD × reading level* interactions on CSF biomarkers

SCD did not interact with reading level on any CSF biomarkers (*p*‐values >0.51). In stratified analyses, SCD was associated with Aβ_42_ (*β* = −5.70, *p* = 0.001) and Aβ_42/40_ (*β* = −0.005, *p* = 0.004) in the higher reading‐level group, but not in the lower reading‐level group (*p*‐values >0.12). These results persisted after FDR correction and were largely unchanged in sensitivity analyses excluding outliers (see Table S1). See Table 3 for results and Figure 2 for illustrations.

### *SCD × education* interactions on CSF biomarkers

SCD interacted with education on Aβ_42_ (*β* = −0.72, *p* = 0.005) and Aβ_42/40_ (*β* = −0.0009, *p* = 0.001), but not on any other CSF biomarkers (*p*‐values >0.23). While the association between SCD and amyloid markers became stronger in individuals of higher education when education was considered as a continuous variable, there were no significant associations when results were stratified by educational tertile. These results persisted after FDR correction and were largely unchanged in sensitivity analyses excluding outliers (see Table S1). See Table 3 for results and Figure 2 for illustrations.

## Discussion

Among community‐dwelling, nondemented older adults, higher levels of SCD were associated with decreased CSF levels of Aβ_42_ and a lower Aβ_42/40_ ratio. SCD was not associated with other CSF biomarkers of tauopathy or neurodegeneration. SCD interacted with cognitive status, sex, and education on CSF Aβ_42/40_ such that associations were stronger in cognitively unimpaired participants, men, and individuals of higher educational levels and educational quality. Taken together, these results highlight the relevance of SCD for screening nondemented older adults for AD pathological changes and suggest that current objective cognitive status and demographic variables are important to consider when doing so.

Our findings add to the growing body of evidence supporting a link between SCD and amyloid deposition in non‐demented older adults,^10^, ^23^, ^24^, ^25^, ^26^ and also demonstrate the clinical utility of a novel measure of SCD. The accumulation of cerebral amyloid is an early pathological event in AD, but its direct association with cognition has been questioned. Given the large number of cognitively normal individuals with evidence of amyloid pathology at autopsy,^27^ it has been assumed that amyloid is not directly associated with cognitive deficits in the absence of tau pathology. In vivo studies using amyloid PET imaging have also demonstrated no significant differences between amyloid‐positive and ‐negative cognitively unimpaired individuals on objective neuropsychological measures.^28^ While individual studies inconsistently observe cognitive impairment in amyloid positive preclinical AD patients, subtle cognitive deficits associated with amyloid pathology have been demonstrated in meta‐analyses.^29^, ^30^ It is possible that the subtle effects of amyloid on cognition are not consistently detectable by objective neuropsychological instruments. Measures of SCD are thought to be elevated at the earliest stages of AD^31^ and may be more attuned toward the subtle changes associated with amyloid accumulation. Beyond amyloid, we found that SCD is not associated with other biomarkers of tauopathy or neurodegeneration. These markers are typically associated with more significant cognitive decline and are closely linked to disease progression to MCI and dementia.^32^ As AD progresses, patients lose insight into their deficits, a phenomenon known as anosognosia. This loss of awareness limits the utility of self‐reported cognitive changes and may explain why SCD was not associated with these biomarkers of more advanced disease.

Indeed, when examining interactions between SCD and cognitive status on CSF biomarkers of AD, we found that the strong associations between SCD and CSF amyloid levels was driven by individuals who were cognitively unimpaired, while there were no significant associations in individuals with MCI. These findings suggest that an individual's subjective report of their cognition is less informative regarding underlying pathologies when the individual has objective cognitive impairment. Past work has shown that self‐reported cognitive complaints (such as the SCD measure used in this study) are accurate at predicting cognitive decline in individuals who are cognitively unimpaired but are less predictive of cognitive decline in individuals with MCI.^33^ At later disease stages, it may be more beneficial to utilize an informant‐reported SCD measure rather than relying on self‐report.

We also found that associations between this novel SCD measure and CSF amyloid levels were varied across sex, with significant associations only being observed in men. This finding is surprising and contrary to past literature which suggests that SCD is more associated with clinical decline in women than men.^8^ We must acknowledge the small sample size of women compared to men completing lumbar puncture in this cohort, thus increasing the risk of Type II error. These analyses should be replicated in larger cohorts to determine the accuracy of these findings. Additionally, women in this sample had lower levels of amyloid and a higher absolute frequency of MCI (though not a statistically significant difference); these differences could be the primary explanation for the observed sex interaction. However, there are a number of potential alternative explanations for this finding. First, men generally are less likely to report or they tend to under‐report the severity of cognitive symptoms.^34^ The current findings could suggest that when men endorse SCD, these reports are more accurately reflecting underlying pathology and amyloid deposition compared to women. These findings could also represent a resilience to amyloidosis in women in the early stages of disease that is not present in men; however, this would be contrary to past work suggesting that women display greater clinical symptoms compared to men with similar levels of pathology.^35^ Similarly, as a group, men had higher levels of education than women in this cohort. However, this was statistically adjusted for and is not likely to fully explain this finding.

Lastly, older women are more likely to experience multiple health problems than men,^36^ and multimorbidity is linked to worse cognitive functioning.^37^ It is possible that SCD is more likely to be related to alternative etiologies other than AD in women than in men.

Further, we found that SCD was more strongly associated with amyloidosis in individuals with greater quantity and quality of education. These findings are consistent with past work which has shown that SCD is more associated with objective cognitive impairment^7^ and development of dementia^38^, ^39^, ^40^ in individuals of higher educational level and the association between SCD and amyloid deposition as seen on PET scans is stronger in more educated older adults.^41^ The current findings extend past work by suggesting the association between education with SCD and biomarker status exists regardless of educational metric (years of education vs. education quality). Given the high level of education attainment in this, and many other, cohorts, future research should examine the effect of educational quality in individuals with fewer years of education. Taken cumulatively, SCD in men and individuals with higher education attainment/quality appear more associated with amyloid accumulation.

This study has a number of strengths. As discussed above, this study utilized a novel SCD measure which has shown excellent psychometric properties, thereby increasing the ability to detect meaningful clinical changes. We utilized a well‐characterized cohort and comprehensively assessed potential confounders. Further, we used core laboratories to analyze CSF using excellent quality control procedures with technicians blinded to clinical information. There were some limitations worth discussion as well. First, the cross‐sectional nature of this study limits the ability to assess causality. Also, after performing an FDR correction, some findings were attenuated. This raises the possibility of false‐positive findings and highlights the necessity of replicating these findings. The sample size is small, particularly for the group of women, which could reduce our ability to detect associations. Finally, this cohort was ethnically/racially homogenous, relatively healthy, and highly educated, thus limiting the generalizability of findings in diverse populations. This homogeneity, particularly in regards to educational attainment and quality, increases the likelihood of false‐negative errors in this study and may have led us to underestimate the impact of education on the association between SCD and CSF biomarkers. Further work is needed to understand the effect of cognitive status and demographic variables on the association between SCD and CSF biomarkers in individuals of diverse sociocultural backgrounds.

In sum, we demonstrated that this novel SCD measure is significantly associated with changes in CSF amyloid in nondemented older adults, with associations being stronger in men and in individuals with higher educational levels. These findings highlight the utility of self‐report measures of SCD in older adults and provide some guidance as to which patient populations may be at greater risk of underlying AD pathology, thereby further advancing personalized medicine in AD and dementia care. Future research is needed to better understand the causes of sex and education‐level differences in the association between SCD and AD to improve screening for early pathological changes across all patient populations.

## Author Contributions

C.J.B. and K.A.G.: conception and design of the study, acquisition and analysis of data, or drafting a significant portion of the manuscript or figures. O.A.K., D.L., S.W., L.D., A.P., K.B., H.Z., T.J.H., and A.L.J.: acquisition and analysis of data or drafting a significant portion of the manuscript or figures.

## Funding Information

This work was supported by K23‐AG045966 (KAG), R01‐AG062826 (KAG), R01‐AG073439 (LCD), F32‐AG076276 (CJB), T32‐AG058524 (CJB), IIRG‐08‐88733 (ALJ), R01‐AG034962 (ALJ), K24‐AG046373 (ALJ), UL1‐TR000445, and UL1‐TR002243 (Vanderbilt Clinical Translational Science Award). HZ is a Wallenberg Scholar supported by grants from the Swedish Research Council (#2022‐01018 and #2019‐02397), the European Union's Horizon Europe research and innovation programme under grant agreement No 101053962, Swedish State Support for Clinical Research (#ALFGBG‐71320), the Alzheimer Drug Discovery Foundation (ADDF), USA (#201809‐2016862), the AD Strategic Fund and the Alzheimer's Association (#ADSF‐21‐831376‐C, #ADSF‐21‐831381‐C, and #ADSF‐21‐831377‐C), the Bluefield Project, the Olav Thon Foundation, the Erling‐Persson Family Foundation, Stiftelsen för Gamla Tjänarinnor, Hjärnfonden, Sweden (#FO2022‐0270), the European Union's Horizon 2020 research and innovation programme under the Marie Skłodowska‐Curie grant agreement No 860197 (MIRIADE), the European Union Joint Programme—Neurodegenerative Disease Research (JPND2021‐00694), the National Institute for Health and Care Research University College London Hospitals Biomedical Research Centre, and the UK Dementia Research Institute at UCL (UKDRI‐1003).

## Conflicts of Interest

Nothing to report.

## Supporting information


Table S1.

## Data Availability

Data may be available upon request at vmacdata.org.

## References

[1] 2021 Alzheimer's disease facts and figures. Alzheimers Dement J Alzheimers Assoc. 2021;17(3):327‐406. doi:10.1002/alz.12328 33756057

[2] Aisen PS , Jimenez‐Maggiora GA , Rafii MS , Walter S , Raman R . Early‐stage Alzheimer disease: getting trial‐ready. Nat Rev Neurol. 2022;4:1‐11. doi:10.1038/s41582-022-00645-6 PMC8978175 35379951

[3] O'Brien K . Screening for cognitive impairment is important and will reduce burdens on our healthcare system. Adv Geriatr Med Res. 2020;2(2):14. doi:10.20900/agmr20200014

[4] Koppara A , Wagner M , Lange C , et al. Cognitive performance before and after the onset of subjective cognitive decline in old age. Alzheimers Dement Diagn Assess Dis Monit. 2015;1(2):194‐205. doi:10.1016/j.dadm.2015.02.005 PMC4876897 27239504

[5] Hohman TJ , Beason‐Held LL , Lamar M , Resnick SM . Subjective cognitive complaints and longitudinal changes in memory and brain function. Neuropsychology. 2011;25(1):125‐130. doi:10.1037/a0020859 20919769 PMC3103103

[6] Kresge HA , Liu D , Khan OA , et al. Subjective cognitive decline is associated with longitudinal cerebral blood flow reductions and gray matter atrophy in older adults. Alzheimers Dement. 2020;16(S6):e043975. doi:10.1002/alz.043975

[7] Wang XT , Wang ZT , Hu HY , et al. Association of subjective cognitive decline with risk of cognitive impairment and dementia: a systematic review and meta‐analysis of prospective longitudinal studies. J Prev Alzheimers Dis. 2021;8(3):277‐285. doi:10.14283/jpad.2021.27 34101784

[8] Heser K , Kleineidam L , Wiese B , et al. Subjective cognitive decline may be a stronger predictor of incident dementia in women than in men. J Alzheimers Dis. 2019;68(4):1469‐1478. doi:10.3233/JAD-180981 30909220

[9] Wolfsgruber S , Jessen F , Koppara A , et al. Subjective cognitive decline is related to CSF biomarkers of AD in patients with MCI. Neurology. 2015;84(12):1261‐1268. doi:10.1212/WNL.0000000000001399 25716354

[10] Wolfsgruber S , Molinuevo JL , Wagner M , et al. Prevalence of abnormal Alzheimer's disease biomarkers in patients with subjective cognitive decline: cross‐sectional comparison of three European memory clinic samples. Alzheimers Res Ther. 2019;11(1):8. doi:10.1186/s13195-018-0463-y 30654834 PMC6337830

[11] Jefferson AL , Gifford KA , Acosta LMY , et al. The Vanderbilt memory & aging project: study design and baseline cohort overview. J Alzheimers Dis. 2016;52(2):539‐559. doi:10.3233/JAD-150914 26967211 PMC4866875

[12] Edmonds EC , McDonald CR , Marshall A , et al. Early vs. late MCI: improved MCI staging using a neuropsychological approach. Alzheimers Dement J Alzheimers Assoc. 2019;15(5):699‐708. doi:10.1016/j.jalz.2018.12.009 PMC6511470 30737119

[13] McKhann GM , Knopman DS , Chertkow H , et al. The diagnosis of dementia due to Alzheimer's disease: recommendations from the National Institute on Aging‐Alzheimer's Association workgroups on diagnostic guidelines for Alzheimer's disease. Alzheimers Dement. 2011;7(3):263‐269. doi:10.1016/j.jalz.2011.03.005 21514250 PMC3312024

[14] Farias ST , Mungas D , Reed BR , et al. The measurement of everyday cognition (ECog): scale development and psychometric properties. Neuropsychology. 2008;22(4):531‐544. doi:10.1037/0894-4105.22.4.531 18590364 PMC2877034

[15] Gilewski MJ , Zelinski EM , Schaie KW . The memory functioning questionnaire for assessment of memory complaints in adulthood and old age. Psychol Aging. 1990;5(4):482‐490. doi:10.1037//0882-7974.5.4.482 2278670

[16] Gass CS , Patten B , Penate A , Rhodes A . The cognitive difficulties scale (CDS): psychometric characteristics in a clinical referral sample. J Int Neuropsychol Soc. 2021;27(4):351‐364. doi:10.1017/S1355617720001058 33081868

[17] Reid LM , Maclullich AMJ . Subjective memory complaints and cognitive impairment in older people. Dement Geriatr Cogn Disord. 2006;22(5–6):471‐485. doi:10.1159/000096295 17047326

[18] Gifford KA , Liu D , Romano R , Jones RN , Jefferson AL . Development of a subjective cognitive decline questionnaire using item response theory: a pilot study. Alzheimers Dement. 2015;1(4):429‐439. doi:10.1016/j.dadm.2015.09.004 PMC4750048 26878034

[19] Palmqvist S , Zetterberg H , Blennow K , et al. Accuracy of brain amyloid detection in clinical practice using cerebrospinal fluid β‐amyloid 42: a cross‐validation study against amyloid positron emission tomography. JAMA Neurol. 2014;71(10):1282‐1289. doi:10.1001/jamaneurol.2014.1358 25155658

[20] Wilkinson GS . WRAT‐3: Wide Range Achievement Test Administration Manual. Western Psychological Services; 1993.

[21] Olsen JP , Fellows RP , Rivera‐Mindt M , Morgello S , Byrd DA . Reading ability as an estimator of premorbid intelligence: does it remain stable among ethnically diverse HIV+ adults? Clin Neuropsychol. 2015;29(7):1034‐1052. doi:10.1080/13854046.2015.1122085 26689235 PMC4738021

[22] Yesavage JA . Geriatric depression scale. Psychopharmacol Bull. 1988;24(4):709‐711. 3249773

[23] Visser PJ , Verhey F , Knol DL , et al. Prevalence and prognostic value of CSF markers of Alzheimer's disease pathology in patients with subjective cognitive impairment or mild cognitive impairment in the DESCRIPA study: a prospective cohort study. Lancet Neurol. 2009;8(7):619‐627. doi:10.1016/S1474-4422(09)70139-5 19523877

[24] Zhao YL , Ou YN , Ma YH , Tan L , Yu JT . Characteristics of subjective cognitive decline associated with Alzheimer's disease amyloid pathology: findings from the CABLE study. J Alzheimers Dis. 2023;92(2):581‐590. doi:10.3233/JAD-221154 36776070

[25] Kim KY , Park J , Jeong YH , et al. Plasma amyloid‐beta oligomer is related to subjective cognitive decline and brain amyloid status. Alzheimers Res Ther. 2022;14(1):162. doi:10.1186/s13195-022-01104-6 36324157 PMC9632136

[26] Miebach L , Wolfsgruber S , Polcher A , et al. Which features of subjective cognitive decline are related to amyloid pathology? Findings from the DELCODE study. Alzheimers Res Ther. 2019;11(1):66. doi:10.1186/s13195-019-0515-y 31366409 PMC6668160

[27] Price JL , Morris JC . Tangles and plaques in nondemented aging and “preclinical” Alzheimer's disease. Ann Neurol. 1999;45(3):358‐368. doi:10.1002/1531-8249(199903)45:3<358::aid-ana12>3.0.co;2-x 10072051

[28] Aizenstein HJ , Nebes RD , Saxton JA , et al. Frequent amyloid deposition without significant cognitive impairment among the elderly. Arch Neurol. 2008;65(11):1509‐1517. doi:10.1001/archneur.65.11.1509 19001171 PMC2636844

[29] Baker JE , Lim YY , Pietrzak RH , et al. Cognitive impairment and decline in cognitively normal older adults with high amyloid‐β: a meta‐analysis. Alzheimers Dement. 2017;6:108‐121. doi:10.1016/j.dadm.2016.09.002 PMC5315443 28239636

[30] Hedden T , Oh H , Younger AP , Patel TA . Meta‐analysis of amyloid‐cognition relations in cognitively normal older adults. Neurology. 2013;80(14):1341‐1348. doi:10.1212/WNL.0b013e31828ab35d 23547267 PMC3656457

[31] Rabin LA , Smart CM , Amariglio RE . Subjective cognitive decline in preclinical Alzheimer's disease. Annu Rev Clin Psychol. 2017;13(1):369‐396. doi:10.1146/annurev-clinpsy-032816-045136 28482688

[32] Khoury R , Ghossoub E . Diagnostic biomarkers of Alzheimer's disease: a state‐of‐the‐art review. Biomark Neuropsychiatry. 2019;1:100005. doi:10.1016/j.bionps.2019.100005

[33] Gifford KA , Liu D , Lu Z , et al. The source of cognitive complaints predicts diagnostic conversion differentially among nondemented older adults. Alzheimers Dement J Alzheimers Assoc. 2014;10(3):319‐327. doi:10.1016/j.jalz.2013.02.007 PMC4064681 23871264

[34] Sundermann EE , Edmonds EC , Delano‐Wood L , et al. Sex influences the accuracy of subjective memory complaint reporting in older adults. J Alzheimers Dis. 2018;61(3):1163‐1178. doi:10.3233/JAD-170425 29332038

[35] Barnes LL , Wilson RS , Bienias JL , Schneider JA , Evans DA , Bennett DA . Sex differences in the clinical manifestations of Alzheimer disease pathology. Arch Gen Psychiatry. 2005;62(6):685‐691. doi:10.1001/archpsyc.62.6.685 15939846

[36] Abad‐Díez JM , Calderón‐Larrañaga A , Poncel‐Falcó A , et al. Age and gender differences in the prevalence and patterns of multimorbidity in the older population. BMC Geriatr. 2014;14:75. doi:10.1186/1471-2318-14-75 24934411 PMC4070347

[37] Wei MY , Levine DA , Zahodne LB , Kabeto MU , Langa KM . Multimorbidity and cognitive decline over 14 years in older Americans. J Gerontol A Biol Sci Med Sci. 2020;75(6):1206‐1213. doi:10.1093/gerona/glz147 31173065 PMC7243582

[38] Geerlings MI , Schmand B , Braam AW , Jonker C , Bouter LM , van Tilburg W . Depressive symptoms and risk of Alzheimer's disease in more highly educated older people. J Am Geriatr Soc. 2000;48(9):1092‐1097. doi:10.1111/j.1532-5415.2000.tb04785.x 10983909

[39] Jonker C , Geerlings MI , Schmand B . Are memory complaints predictive for dementia? A review of clinical and population‐based studies. Int J Geriatr Psychiatry. 2000;15(11):983‐991. doi:10.1002/1099-1166(200011)15:11<983::aid-gps238>3.0.co;2-5 11113976

[40] van Oijen M , de Jong FJ , Hofman A , Koudstaal PJ , Breteler MMB . Subjective memory complaints, education, and risk of Alzheimer's disease. Alzheimers Dement J Alzheimers Assoc. 2007;3(2):92‐97. doi:10.1016/j.jalz.2007.01.011 19595922

[41] Aghjayan SL , Buckley RF , Vannini P , et al. The influence of demographic factors on subjective cognitive concerns and beta‐amyloid. Int Psychogeriatr. 2017;29(4):645‐652. doi:10.1017/S1041610216001502 27724996 PMC5361739

