# Supplementary material for: Cognitive status and demographics modify the association between subjective cognition and amyloid
Source: Ann Clin Transl Neurol. 2024 Oct 23;11(11):2977–86. doi: 10.1002/acn3.52209 (PMC11572737; doi:10.1002/acn3.52209)
Supplement: Supplementary file 1 — Table S1. [file ACN3-11-2977-s001.docx]

| **Supplementary Table 1. SCD and CSF Biomarkers: Interaction and Stratified Models–Sensitivity Analyses Excluding Outliers.** | | | | | | | | | | | | | |
| --- | --- | --- | --- | --- | --- | --- | --- | --- | --- | --- | --- | --- | --- |
|  | β | 95% CI | | *p* | β | 95% CI | | | *p* | β | | 95% CI | *p* |
| **SCD x Diagnosis Interactions** | | | | | *Cognitively Normal (n=72)* | | | | | *MCI (n=43)* | | | |
| Aβ42, pg/mL | 5.75 | 1.48, 10.01 | **0.009*** | | -5.39 | | -8.83, -1.96 | **0.003*** | | 0.35 | -2.73, 3.43 | | 0.82 |
| Aβ42/40 | 0.006 | 0.001, 0.01 | **0.02** | | -0.006 | | -0.01, -0.003 | **0.0005*** | | -0.0009 | -0.006, 0.004 | | 0.69 |
| Tau, pg/mL | -2.21 | -6.24, 1.81 | 0.28 | | 1.70 | | -0.73, 4.13 | 0.17 | | 0.87 | -3.43, 5.18 | | 0.68 |
| P-tau, pg/mL | -0.28 | -0.76, 0.20 | 0.25 | | 0.19 | | -0.11, 0.49 | 0.21 | | 0.04 | -0.46, 0.55 | | 0.87 |
| NfL, pg/mL | 2.97 | -5.64, 11.59 | 0.50 | | -1.32 | | -6.02, 3.38 | 0.58 | | 4.89 | -4.60, 14.37 | | 0.30 |
| **SCD x Sex Interactions** |  | | | | *Men (n=93)* | | | | | *Women (n=36)* | | | |
| Aβ42, pg/mL | 3.11 | -0.59, 6.80 | | 0.10 | -5.11 | -7.94, -2.29 | | | **0.0005*** | 1.59 | | -2.40, 5.58 | 0.42 |
| Aβ42/40 | 0.005 | 0.0006, 0.009 | | **0.03** | -0.006 | -0.009, -0.003 | | | **0.0003*** | 0.002 | | -0.003, 0.007 | 0.48 |
| Tau, pg/mL | -2.27 | -5.48, 0.94 | | 0.16 | 1.78 | -0.39, 3.95 | | | 0.11 | 0.65 | | -4.36, 5.66 | 0.79 |
| P-tau, pg/mL | -0.26 | -0.64, 0.13 | | 0.19 | 0.16 | -0.11, 0.43 | | | 0.25 | 0.06 | | -0.50, 0.63 | 0.82 |
| NfL, pg/mL | -1.76 | -8.85, 5.33 | | 0.62 | 3.35 | -1.08, 7.77 | | | 0.14 | -0.51 | | -12.19, 11.16 | 0.93 |
| **SCD x Reading Level Interactions** | | | | | *Lower Half (n=61)* | | | | | *Upper Half (n=68)* | | | |
| Aβ42, pg/mL | -1.86 | -8.22, 4.50 | | 0.56 | -2.30 | -5.75, 1.15 | | | 0.19 | -5.70 | | -9.10, -2.29 | **0.001*** |
| Aβ42/40 | -0.002 | -0.01, 0.005 | | 0.84 | -0.003 | -0.007, 0.0008 | | | 0.12 | -0.005 | | -0.009, -0.002 | **0.004*** |
| Tau, pg/mL | 1.97 | -3.50, 7.45 | | 1.00 | 0.68 | -1.48, 2.84 | | | 0.53 | 1.63 | | -1.67, 4.92 | 0.33 |
| P-tau, pg/mL | 0.18 | -0.48, 0.84 | | 0.97 | 0.09 | -0.17, 0.35 | | | 0.50 | 0.11 | | -0.29, 0.51 | 0.60 |
| NfL, pg/mL | -0.27 | -12.52, 11.98 | | 0.62 | 0.23 | -6.67, 7.12 | | | 0.95 | 2.78 | | -2.72, 8.29 | 0.32 |
| **SCD x Education Interactions** | | | | | *Lowest Tertile (n=45)* | | | | | *Highest Tertile (n=22)* | | | |
| Aβ42, pg/mL | -0.72 | -1.21, -0.22 | | **0.005*** | 1.25 | -2.32, 4.81 | | | 0.48 | -4.36 | | -10.35, 1.63 | 0.14 |
| Aβ42/40 | -0.0009 | -0.001, -0.0004 | | **0.001*** | 0.002 | -0.002, 0.005 | | | 0.28 | -0.006 | | -0.02, 0.005 | 0.23 |
| Tau, pg/mL | 0.27 | -0.17, 0.70 | | 0.23 | 0.18 | -3.33, 3.70 | | | 0.92 | 1.12 | | -5.68, 7.91 | 0.73 |
| P-tau, pg/mL | 0.03 | -0.02, 0.08 | | 0.24 | 0.03 | -0.37, 0.43 | | | 0.87 | 0.10 | | -0.83, 1.04 | 0.81 |
| NfL, pg/mL | -0.45 | -1.41, 0.51 | | 0.36 | 7.52 | -0.92, 15.95 | | | 0.08 | 0.22 | | -10.66, 11.10 | 0.97 |
| Note. Models were adjusted for age, sex, education, race/ethnicity, *APOE*-ε4 status, cognitive status, and GDS. β Indicates the degree of change in outcomes per 1 unit increase in SCD. Bold font indicates *p*-value<0.05. *FDR-adjusted *p*-value<0.05. Aβ, amyloid beta; APOE, apolipoprotein E; FDR, false discovery rate; GDS, Geriatric Depression Scale; NfL, neurofilament light; p-tau, phosphorylated tau; SCD, subjective cognitive decline. | | | | | | | | | | | | | |
